# Effects of Prefrontal Transcranial Direct Current Stimulation and Motivation to Quit in Tobacco Smokers: A Randomized, Sham Controlled, Double-Blind Trial

**DOI:** 10.3389/fphar.2018.00014

**Published:** 2018-01-26

**Authors:** Maria C. Vitor de Souza Brangioni, Danilo A. Pereira, Aurore Thibaut, Felipe Fregni, Joaquim P. Brasil-Neto, Raphael Boechat-Barros

**Affiliations:** ^1^Hospital Universitário de Brasília, Universidade de Brasília, Distrito Federal, Brazil; ^2^IBNeuro-Instituto Brasileiro de Neuropsicologia e Ciências Cognitivas, Brasília, Brazil; ^3^Spaulding Neuromodulation Center, Spaulding Rehabilitation Hospital and Massachusetts General Hospital, Harvard Medical School, Boston, MA, United States; ^4^Departamento de Ciências Fisiológicas, Instituto de Biologia, Universidade de Brasília, Brasília, Brazil; ^5^Laboratório de Psiquiatria, Faculdade de Medicina, Universidade de Brasília, Brasília, Brazil

**Keywords:** transcranial direct current stimulation (tDCS), smoking, craving, motivation, prefrontal cortex

## Abstract

Transcranial direct current stimulation (tDCS) applied over the dorsolateral prefrontal cortex (DLPFC) has been shown to reduce cravings in tobacco addiction; however, results have been somewhat mixed. In this study, we hypothesized that motivation to quit smoking is a critical factor of tDCS effects in smokers. Therefore, we conducted a double-blind, randomized clinical trial to evaluate the effects of both tDCS and motivation to quit on cigarette consumption and the relationship between these two factors. DLPFC tDCS was applied once a day for 5 days. Our primary outcome was the amount of cigarettes smoked per day. We collected this information at baseline (d1), at the end of the treatment period (d5), 2 days later (d7) and at the 4-week follow-up (d35). Visual Analog Scale (VAS) for motivation to quit was collected at the same time-points. 36 subjects (45 ± 11 years old; 24.2 ± 11.5 cigarettes daily smoked, 21 women) were randomized to receive either active or sham tDCS. In our multivariate analysis, as to take into account the mediation and moderation effects of motivation to quit, we found a significant main effect of tDCS, showing that tDCS was associated with a significant reduction of cigarettes smoked per day. We also showed a significant interaction effect of motivation to quit and treatment, supporting our hypothesis that tDCS effects were moderated by motivation to quit, indicating that higher levels of motivation were associated with a larger tDCS response. We found that the participants' motivation to quit alone, both at baseline and at follow-up, does not explain the decrease in the average cigarette consumption. Repetitive prefrontal tDCS coupled with high motivation significantly reduced cigarette consumption up to 4-weeks post-intervention.

Clinical Trial Registration: http://ClinicalTrials.gov, NCT02146014.

## Introduction

Smoking is the leading cause of preventable death worldwide and has been included in the International Classification of Diseases (ICD) of the World Health Organization (WHO) since 1992. It is considered to be a pandemic with one-third of the world population smoking, and is estimated to be the cause of more than 5 million deaths globally each year (Bennett, 2016). Like other dependences, addiction to nicotine is a progressive, chronic, recurrent disorder mediated by action on central and peripheral nicotinic receptors, being supported by environmental, biological and psychological factors (Longo et al., 2016). It is a very complex disease for which treatment is still a challenge. Although 70% of smokers would like to quit (Lader and Goddard, 2005), only approximately 30–40% of them actually attempt to quit (Cokkinides et al., 2005; West et al., 2005). However, <5% of smokers reach their goal and succeed in long-term smoking cessation (Royal College of Physicians, 2000).

One of the reasons for the low rate of quitting is the potent effect of nicotine on the reward system. The nicotine induced rewarding effect is probably mediated by the dopaminergic mesocortico-limbic system and its projections from the ventral tegmental area (VTA) to the *nucleus accumbens* (NAC) and to the prefrontal cortex (PFC). This system forms a cerebral gratification circuit with dopamine as the neurotransmitter, determining the sensation of pleasure associated with smoking (Koob and Volkow, 2010). Mansvelder and McGehee (2002) demonstrated that in addition to increasing dopamine release, nicotine also induces a prolonged increase in glutamatergic excitatory activity and a reduction in GABAergic inhibitory activity on the mesocortico-limbic pathway (Mansvelder and McGehee, 2002). This pathway is related to the associated inability to voluntarily reduce drug use despite potentially catastrophic consequences (Koob and Le Moal, 2008). Nicotine craving is one of the most prominent symptoms and considered the greatest obstacle to quitting smoking (Wray et al., 2013). Therefore, it should be given particular attention as a potential target to treat and reduce smoking. Recent neuroimaging studies evidenced that the dorsolateral prefrontal cortex (DLPFC) is a critical component of the neural substrate for craving associated with various psychoactive substances (Hartwell et al., 2011). More specifically, for smokers, it is thought to underlie the cognitive control of craving and reward (Goldstein and Volkow, 2011).

Indeed, techniques of neuromodulation targeting the prefrontal cortex can be used to reduce smoking and related behaviors. Previous transcranial Direct Current Stimulation (tDCS) studies have demonstrated the effectiveness of this technique in reducing the desire for smoking in active smokers (Fregni et al., 2008a; Boggio et al., 2009; Falcone et al., 2016). However, results are mixed and the main reason is that other factors may mediate the effects of tDCS on cigarette consumption or craving.

In the present study, we evaluated the direct effects of tDCS and motivation to quit smoking on the participants' average cigarette consumption. We also aimed to evaluate if the decrease in cigarette consumption is caused by tDCS alone, by the participants' motivation to stop smoking or by the interaction between tDCS and motivation.

Our primary outcome was the reduction of cigarette consumption at 4 weeks post-intervention to evaluate the long-term effects of tDCS.

## Methods

### Study design

We conducted a parallel phase II randomized, double blind, controlled clinical trial. Participants were required to meet the following inclusion criteria: age ranging from 18 to 65 years-old, being an active smoker (i.e., at least 10 cigarettes per day) for at least 1 year, and being able to provide informed consent to participate. Exclusion criteria were as follows: being pregnant, having important clinical or psychiatric comorbidity that could interfere with the follow-up, being illiterate, or being treated for smoking at the time of enrollment.

The present study was approved by the Ethics Committee on Research in Human Beings of the Faculty of Health Sciences at the University of Brasília (CEP/FS/UnB), registry N: 080/10, deposited in the Registry System http://ClinicalTrials.gov under the number NCT02146014. All subjects participated voluntarily and signed the Informed Consent Form. The sample was recruited through flyers at the University of Brasília's Hospital (HUB), via dissemination on the university's website and local print media.

Thirty-six smokers participated in the study (mean age: 45 ± 11 years old; mean duration of cigarette consumption: 28.2 ± 11.3 years; average cigarettes per day: 24.2 ± 11.5; 21 women). Note that no sample size calculation was performed.

### Procedures

**Figure 1:**
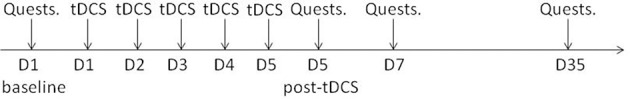
Study protocol. Participants received either 5 days of active tDCS or 5 days of sham tDS. Questionnaires were collected at baseline, after 5 days of tDCS, 2 days later (day 7) and 4 weeks later (day 35).

This trial had two arms, active and sham tDCS, and the protocol consisted of a total of 7 visits. Each participant received active or sham tDCS once a day for five consecutive days (day 1 to day 5). A computer-generated randomization sequence was used to assign the group, active or sham, in a 1:1 allocation ratio. Assessements were performed at baseline (d1), at the end of the stimulation sessions (d5), 2 days later (d7) and at 4 weeks follow-up (day 35); see Figure 1.

A battery-powered direct current stimulator which uses two 9-Volt batteries for current generation and an additional 9-volt battery for an analog amperemeter display was used. The device has a sham mode switch and is capable of delivering from 0 to 4 mA through a pair of conductive rubber electrodes (anode and cathode) fitted with saline-soaked sponges.

Electrodes coated with sponges, measuring 35 cm^2^ (7 × 5 cm), soaked in saline solution were used with the following montage: anode over the left DLPFC (area F3 according to the 10–20 international system) and cathode over the right contralateral supraorbital region.

During the experiment, subjects were sitting comfortably in an armchair in a bright and quiet room. The device was placed strategically behind the armchair, hidden from the patient during the stimulation. A constant current of 1 mA for 20 min was applied with a 10-s ramp-up at the beginning and a 10-s ramp-down at the end of the stimulation. For the sham tDCS, the same electrode montage was used with the same ramps-up and down of 10 s; however, the current was turned off after 20 s. A third person, not involved in the assessments or in data analysis, applied the stimulation. This procedure allowed both patients and assessors to be blinded to the treatement allocation.

### Outcomes

Our primary outcome was the amount of cigarettes smoked per day at the 4-week follow-up (d35). A self-monitoring questionnaire to measure the number of cigarettes smoked during the protocol was asked at baseline, at the end of the stimulation sessions (d5), 2 days later (d7) and at the 4-week follow-up (d35).

As secondary outcome measures, each participant completed the following questionaires: (1) Structured Clinical Interview for DSM-IV SCID-I Axis Disorders 1 (Del-Ben et al., 2001); (2) questionnaire with sociodemographic and clinical data; (3) questionnaire on the history of smoking; (4) Fagerström's Nicotine Dependence Test (FNDT) (Heatherton et al., 1991); (5) Visual Analog Scale (VAS—ranging from 0 to 10) of willigness or desire to smoke a cigarette; (6) VAS for the motivation to quit smoking. All procedures were performed in the hospital. These questionnaires were completed at baseline, at the end of the 5 days of stimulation, and also at the 1 and 4-week follow-ups.

Baseline characteristics (FNDT, initial cigarette consumption, motivation to quit, age, gender, hand laterality), were compared between groups and reported in Supplementary Table 1.

### Statistical analyses

Baseline characteristics between the two groups (active and sham) were compared for the nominal and ordinal data using a chi-square test (likelihood ratio).

**Figure 2:**
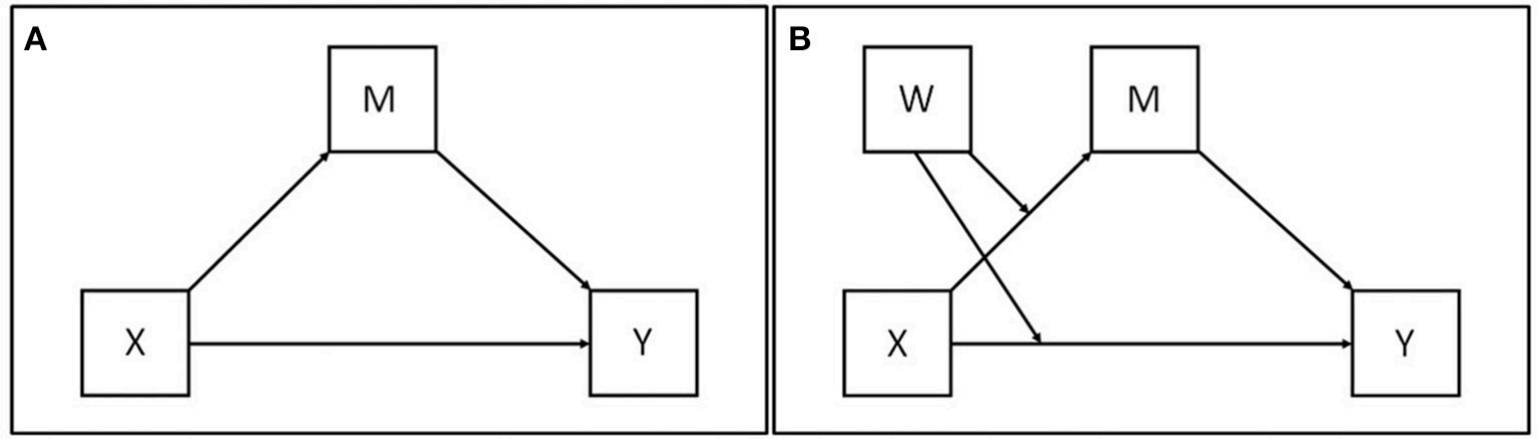
Mediator model. **(A)** Simplest model, two consequential variables (M) and (Y) and two antecedent variables (X) and (M), with X causally influencing Y and M, and M causally influencing Y (Hayes, 2013). **(B)** Conditional model of mediation and moderation, where it depicts the mediation of the effect of X on Y through M, with both direct and indirect effects of X moderated by W.

To analyze how subjects' motivation to quit interacts with the clinical intervention (i.e., tDCS), multivariate linear regressions were used. Mediation analysis is a statistical method used to help answer questions of how a causal agent X conveys its effect on the outcome variable Y (see Figure 2A; for more details on the statistical approach see Hayes, 2013). Figure 2B represents a conditional model of mediation and moderation, where it depicts the mediation of the effect of X on Y through M, with both direct and indirect effects of X moderated by W. The moderation of the indirect effect is portrayed as a result of moderation of the effect of X on M through W. This moderation processes the indirect effect conditioned to W. The direct effect is also proposed as moderated by W, such that the direct effect is also a condition of W. Thus, there is no single direct or indirect effect of X on Y. Instead, the direct and indirect effects are functions of W. For more details regarding the statistical approach, see Hayes (2013). In this study, the mediation analysis has two linear regressions, where the *treatment* is the binary intervention variable (1 = active tDCS, 0 = sham tDCS) and *m1d* is the motivation of the subject to stop smoking at baseline. The variable *m1d* is used in the analysis to strengthen the statistical power in detecting the treatment effect. It also serves to explore the hypothesis of interaction at the baseline between the *treatment* intervention and the *m1d* motivation, called here as *treatmentXm1d*. The covariant *m1d* is referred to as a moderator and is measured before treatment; this variable/it is not correlated with treatment due to randomization of subjects. The variable *m35d* is the subject's motivation to stop smoking at d35 of the study. This variable is considered as a mediator in the model. The outcome variable is the average number of cigarettes smoked per day, reported at d35 of the survey, *cigar35d*. This variable does not violate the temporal order of events between the mediator variable (*m35d*) and the outcome variable (*cigar35d*).

The following regression equations can express the model:

(1) $$\begin{array}{l} cigar35d_{i}=\beta_{0}+\beta_{1}m35d+\beta_{2}treat_{i}+\beta_{3}m1d\\ \;+\beta_{4}treatXm1d_{i}+\varepsilon_{1\text{i}}, \end{array}$$

(2) $$\begin{array}{l} m35d_{i}=\gamma_{0}+\gamma_{1}treat_{i}+\gamma_{2}m1d_{i}+\gamma_{3}treatXm1d_{i}+\varepsilon_{2i}, \end{array}$$

(3) $$\begin{array}{l} =\gamma_{0}+(\gamma_{1}+\gamma_{3}m1d_{i})treat_{i}+\gamma_{2}m1d_{i}+\varepsilon_{2i}, \end{array}$$

Inserting Equation (3) in Equation (1) results:

(4) $$\begin{array}{l} cigar35d_{i}=\beta_{0}+\beta_{1}\gamma_{0}+\beta_{1}\left(\gamma_{1}+\gamma_{3}m1d_{i}\right)treat_{i}\\ +\beta_{1}\gamma_{2}m1d_{i}+\beta_{1}\varepsilon_{2\text{i}}, \end{array}$$

(5) $$\begin{array}{l} +\beta_{2}treat_{i}+\beta_{3}m1d_{i}+\beta_{4}treat_{i}Xm1d_{i}+\varepsilon_{1\text{i}}. \end{array}$$

It can be observed in the first line of Equation (4) that the indirect effect of treatment on *cigar35d* is β_1_ (γ_1_ + γ_3_ *m1di*), where *m1d* moderates the treatment effect. It is observed in the second line that the direct effect of treatment on *cigar35d* is β_2_ + β_4_ *treat*.

As sub-analysis, we performed the same tests dividing participants by smoking frequency: below average, average and above average.

Missing data were treated as missing at random (MAR) using the multiple imputation method FIML (full information maximum likelihood) in Mplus software.

All statistical analyses were performed using the statistical program Mplus (version 7.4). The syntax of Mplus was obtained from suggestions from session 2.6.5 of Muthén, Muthén and Asparouhov (2016).

## Results

Out of the 36 participants, 19 were allocated to the active tDCS group and 17 received sham-tDCS. All 36 smokers completed the 5-day tDCS application protocol according to their allocation group (see Supplementary Figure 1).

No side effects were observed during or after the applications, with no complaints of pain or discomfort.

Active and sham groups were homogeneous in relation to age (younger and older than 40 years old), gender, laterality, psychiatric comorbidities (as assessed by the Structured Clinical Interview for DSM-IV Axis I Disorders—SCID-I), fear of gaining weight, whether they were seriously thinking about quitting smoking, motivation to quit smoking altogether and whether they had quit smoking previously. Fagerström scale questions did not differ between groups either (all *p*s > 0.05). Patients' characteristics and differences between groups (active vs. sham) can be found in Supplementary Table 2.

In the multivariate analysis (including motivation to stop smoking (at d1 and d35), treatment and interaction [motivation (d1) vs. treatment)], we found that the mean number of cigarettes consumed is significantly influenced by the *treatment* variable (*p* = 0.033). As the treament variable is dichotomous [1 = treatment, 0 = placebo], the regression can also be interpreted as a difference between the means of cigarette consumption between the two groups; subjects who received active tDCS consumed on average 7.11 cigarettes less than the sham group, when adjusted to these variables. tDCS induced decrease in cigarette consumption was modified by the level of motivation to quit at baseline [*p* = 0.032, interaction term between the intervention and the motivation at the baseline (*treatXm1d*)]. However, we observed that the main effect of motivation to quit smoking, recorded both in the baseline (*m1d*) and in the final phase (*m35d*), did not influence the average final cigarette consumption alone (*cigar35d*) (*p* = 0.073 and *p* = 0.469, respectively). In addition, baseline motivation to stop smoking did not affect motivation at 4-week follow-up (*p* = 0.108), which did not differ significantly between treatment and placebo groups (*p* = 0.201). All results are presented in Table 1.

**Table 1:** Moderate mediation regression of the randomized intervention to reduce the average cigarette consumption per day with treatment with the interaction of the treatment with the motivation at the baseline using a robust likelihood estimator (MLR).

	**Variable**	**Raw coeff**.	**S.E**.	***z* (est./S.E.)**	***p*-value**	**StdY. coeff**.	**S.E**.	***z* (est./S.E.)**	***p*-value**
N cigar consumption (35th day)	m35d	−1.12	1.54	−0.72	0.469	−0.21	0.29	−0.72	0.474
	treat	−7.11	3.34	−2.13	0.033^*^	−0.71	0.32	−2.23	0.026^*^
	m1d	2.33	1.30	1.79	0.073	0.23	0.12	1.88	0.060
	treatXm1d	−2.86	1.33	−2.15	0.032^*^	−0.28	0.13	−2.28	0.023^*^
Motivation to quit (35th day)	treat	−0.64	0.50	−1.28	0.201	−0.34	0.26	−1.31	0.190
	m1d	0.29	0.18	1.61	0.108	0.16	0.10	1.66	0.098
	treatXm1d	0.31	0.20	1.56	0.118	0.17	0.11	1.49	0.136
Intercepts	m35d	8.99	0.35	25.69	0.000^*^	4.86	0.80	6.07	0.000^*^
	cigar35d	26.96	13.87	1.94	0.052	2.68	1.37	1.96	0.050^*^
Residual variances	m35d	1.61	0.58	2.79	0.005^*^	0.47	0.15	3.23	0.001^*^
	cigar35d	70.14	15.96	4.40	0.000^*^	0.70	0.16	4.32	0.000^*^
New/Additional	ind_low	1.62	2.59	0.62	0.533				
	ind_avg	0.71	1.26	0.56	0.574				
	ind_high	−0.20	0.55	−0.36	0.721				
	dir_low	0.40	5.22	0.08	0.939				
	dir_avg	−7.11	3.34	−2.13	0.033^*^				
	dir_high	−14.63	4.42	−3.31	0.001^*^				
*R*^2^	m35d	0.53	0.15	3.65	0.000^*^				
	cigar35d	0.31	0.16	1.89	0.058				

*The values on the left refer to the raw scores (non-standardized), and the values on the right to the outcome variable standardized scores*.

* *p < 0.05; est., estimation; S.E., standard error; z (est./S.E.), effect size; R^2^, coefficient of multiple determination for multiple regression*.

When adding the variable number of cigarettes smoked per day (below, on average and above average) into the regression, we observed that only the *average* (*dir_avg*, with average cigarette consumption) and *higher* (*dir_high*, with cigarette consumption above average [+1 DP)] were significant (*p* = 0.033 and *p* = 0.001, respectively). The treatment was not effective for participants who had below-average cigarette consumption (*dir_low, p* = 0.939) when compared to placebo.

The *R*^2^ values in Table 1 show the explanatory capacity of the model. In other words, it shows how independent variables share or explain the variability of the dependent variable. Considering that the consumption of cigarettes on the 35th day can be explained by direct and indirect effects, all the variables that make up the model account for 31% of its variability. Considering only the motivation to quit at d35 (*m35d*), 53% of the model variability can be explained by the motivation to stop smoking at baseline (*m1d*), by the treatment (i.e, active tDCS) and by the interaction between both variables (m1d and active tDCS).

**Figure 3:**
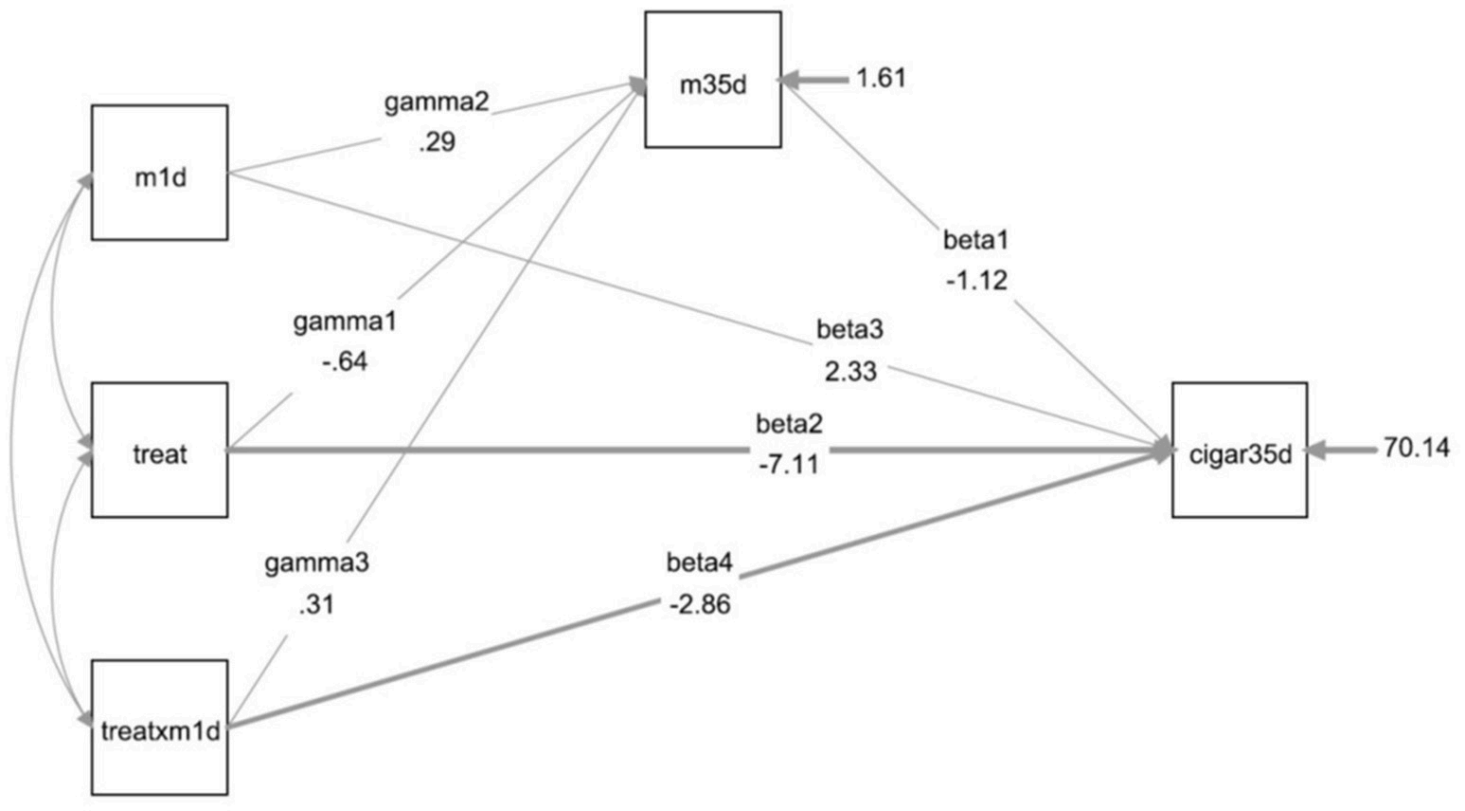
Statistical model of the moderate mediation analysis of a randomized intervention (treatment vs. placebo) to reduce the average number of cigarettes consumed per day (*cigar35d*) with the interaction (*treatXm1d*) between treatment (*treat*) and motivation (*m1d*) in the 1st day of the experiment (baseline). The thicker arrows (β_2_ and β_4_) represent significant coefficients of the model.

Figure 3 shows the statistical diagram of the model presented in Table 1.

## Discussion

In this study, we confirmed our hypothesis that the effect of tDCS on cigarette consumption is partially influenced by motivation to quit. In fact, we found that tDCS effect arises from a conditional analysis of mediation and moderation, where the treatment effect is mediated by the participant's motivation to stop smoking, with both direct and indirect effects. When adding restrictions to the model, we found that the direct effect of the treatment mediated by the participant's motivation to quit appears to be significant only among those who had a cigarette consumption within or above average (>7 cigarettes per day); this significance was not observed among the participants with consumption below average (≤7 cigarettes per day). That is, the treatment did not directly benefit those who smoked less cigarettes than average.

Previous tDCS studies aiming at treating smokers targeted the dorsolateral prefrontal cortex (DLPFC), which is thought to be involved in the cognitive control of craving and reward related to smoking (Goldstein and Volkow, 2011). So far, four studies have investigated the effects of prefrontal tDCS in active smokers aiming at reducing either cigarette consumption or craving. A single tDCS session has shown to reduce smoking craving (Fregni et al., 2008a), while 5 days of stimulation induced a reduction of craving and a 30% decrease in cigarette consumption, showing the potential clinical effects of tDCS in managing smoking behaviors (Boggio et al., 2009). In addition, prefrontal tDCS applied for 10 days influenced decision making behaviors associated with a significant reduction of cigarettes consumed (Fecteau et al., 2014). Recently, a single session of tDCS demonstrated a reduction in the latency to smoke, highlighting the effect of tDCS on patients' ability to resist smoking (Falcone et al., 2016). Another study targeted the fronto-parieto-temporal association area aiming to reduce smoking-related behaviors (Meng et al., 2014). However, no significant effects on smoking behavior have been found. The above studies suggest that tDCS applied over the left DLPFC can help smokers in smoking cessation or reduction. Besides reducing smoking craving behaviors and consumption, prefrontal tDCS has also been found to lessen craving for food and alcohol (Boggio et al., 2008; Fregni et al., 2008a,b; Goldman et al., 2011), as well as modulate behaviors related to addictive disorders such as risk-taking and impulsivity (Fecteau et al., 2007a,b; Beeli et al., 2008; Boggio et al., 2010; Batista et al., 2015), highlighting the positive effects of prefrontal tDCS on reducing craving for various addictive disorders.

Neuroimaging studies have shown that active smoking induces a decrease in gray matter volume (i.e., voxel based morphometry) of the DLPFC (Brody et al., 2004; Gazdzinski et al., 2005; Gallinat et al., 2006). A correlation between duration of smoking and gray matter reduction in this area was also identified (Gallinat et al., 2006). Other MRI studies confirmed this relationship, showing that a decrease in gray matter density in the DLPFC correlated with longer exposure to cigarettes (Nestor et al., 2011; Zhang et al., 2011). Therefore, it is conceivable that an increase in the excitability of this brain region, by means of tDCS, may help to revert these neural maladaptive changes or slowdown these processes.

It has been suggested that the after effects of tDCS are mediated through the modulation of N-methyl-D-aspartic acid (NMDA) (Nitsche et al., 2003) and DA-D2 receptor activation (Nitsche et al., 2006). As proposed by Wing et al. (2013), tDCS over the DLPFC can lead to neuronal excitability correlated with dopamine and GABA neurotransmitters, among others, in cortical and subcortical brain regions (Wing et al., 2013). These effects could explain the observed behavioral improvements in smoking habits such as increased inhibitory control, improved decision-making and decreased craving which ultimately may reduce cigarette consumption.

In fact, the interaction between tDCS effects and motivation levels support the notion that the main effect of tDCS was mediated by higher order cognitive functioning. Subjects with higher levels of motivation to quit at day 1 seem to have had a larger cigarette reduction compared to those with less motivation to stop smoking. These results go along with the main mechanism of tDCS: by enhancing spontaneous neuronal firing, tDCS can have an impact on behaviors that the subject is engaged in (in this case, motivation to quit smoking). In a recent study assessing the influence of financial motivation on tDCS effects on working memory performance in subjects with low working memory capacity (who did not improve following tDCS), showed that increasing external motivation restored tDCS-related benefits on working memory (Jones et al., 2015). This result highlights the importance of the participant's motivation to achieve a goal on tDCS-related effects. In other words, a subject who is not motivated to perform a task, with or without tDCS, has less chance to improve following tDCS. Some could argue that motivation to quit alone (without any other treatment or therapy) is sufficient to reduce cigarette consumption. However, in the present study, motivation to quit at day 1 and day 35, taken individually, did not explain the observed reduction in cigarette consumption. On the other hand, the interaction between motivation to quit and active treatment (i.e., tDCS) had a significant impact on the number of cigarettes smoked per day.

As for other conditions, tDCS can induce clinical changes for impaired behaviors. For instance, tDCS can enhance hand motor function when applied over the non-dominant hemisphere but not when the dominant motor cortex is stimulated (Boggio et al., 2006). As aforementioned, cigarette consumption induces gray matter changes, including the prefrontal cortex. Therefore, for smokers consuming below average cigarettes, the pathological changes may not have occurred yet and the neurophysiological effects of tDCS would, as a consequence, be distinct, which could explain why the effects of tDCS were not observed in this subgroup of patients. It is also important to take into account that nicotine addiction withdrawal has been found to lead to a reduction of neuroplasticity (Grundey et al., 2012); this decrease in plasticity during the quitting period may be an obstacle to smoking cessation and tDCS effects. Nicotine influences the dopaminergic system, as well as nicotinic acetyl-choline receptors (nAChRs) and the adrenergic, serotonergic, glutamatergic, and GABAergic systems (Levin et al., 2006), which can limit tDCS effects as its mechanisms involve similar plastic pathways (Nitsche and Paulus, 2000, 2001). For this reason, it is essential to take these mechanisms of neuroplasticity reduction into account when designing a protocol, with special attention to the timing of stimulation as it may be critical.

It should be noted that participants were not asked to guess which treatment they received after the stimulation sessions. However, using 30 s of stimulation, which induces the initial itching sensation, is a reliable method of blinding as shown by a previous randomized controlled study using the same stimulation parameters (Fregni et al., 2005).

Future studies should combine prefrontal tDCS with behavioral treatments to increase motivation to stop smoking or taking an addictive substance, since based on our findings, both tDCS and high motivation to quit are necessary to significantly reduce tobacco consumption. It is now widely accepted that tDCS effects can be manipulated by subject's state (Silvanto et al., 2007; Silvanto and Pascual-Leone, 2008); by combining motivational approaches with repeated tDCS sessions (e.g., for 4 weeks) applied over the DLPFC, tDCS could be an adjuvant treatment to help smokers to quit. Long-term evaluations to assess the remission rate are also required.

## Author contributions

All authors listed have made a substantial, direct and intellectual contribution to the work, and approved it for publication.

### Conflict of interest statement

The handling editor declared a shared affiliation, though no other collaboration, with authors AT and FF. The authors declare that the research was conducted in the absence of any commercial or financial relationships that could be construed as a potential conflict of interest.
