# Supplementary material for: Effects of Prefrontal Transcranial Direct Current Stimulation and Motivation to Quit in Tobacco Smokers: A Randomized, Sham Controlled, Double-Blind Trial
Source: Front Pharmacol. 2018 Jan 26;9:14. doi: 10.3389/fphar.2018.00014 (PMC5791546; doi:10.3389/fphar.2018.00014)
Supplement: Supplementary file 1 [file Table1.docx]

|  | Treatment vs. placebo groups | Likelihood ratio | d.f. | p |
| --- | --- | --- | --- | --- |
| General | Age group (< 40 or > 39 yrs.) | .056 | 1 | .813 |
|  | Gender (Male vs Female) | .003 | 1 | .955 |
|  | Previous attempts to quit smoking | 1.96 | 3 | .580 |
|  | Hand laterality | .122 | 1 | .726 |
|  | Psychiatric comorbidities | 1.84 | 1 | .175 |
|  | Fear of gaining weight if you stop smoking | .003 | 1 | .955 |
|  | Seriously thinking about quitting smoking | 3.19 | 5 | .527 |
|  | How motivated you are to stop smoking completely? | 2.11 | 2 | .348 |
|  | Have you ever stopped smoking before? | .056 | 1 | .813 |
| Fargestron | Question #1 | .623 | 3 | .960 |
|  | Question #2 | .000 | 1 | .983 |
|  | Question #3 | .551 | 1 | .458 |
|  | Question #4 | 6.70 | 3 | .082 |
|  | Question #5 | .146 | 1 | .702 |
|  | Question #6 | .185 | 1 | .667 |

Table s1 – Contingency table tests showing group differences between baseline characteristics.

| **Demographic data** | **Sham tDCS  (n=17)** | **Active tDCS (n=19)** | **P-value** |
| --- | --- | --- | --- |
|  |  |  |  |
| **Handedness*** |  |  |  |
| Right | 15 (88,2%) | 16 (84,2%) | 0.727 |
| Left | 2 (11,8%) | 3 (15,8%) |  |
| **Clinical comorbidities***** |  |  |  |
| No | 7(41,2%) | 11(57%,9%) | 0.255 |
| Yes | 10(58,8%) | 8(42,1%) |  |
| **Psychiatric comorbidities (SCID)*^(1)^** |  |  |  |
| No | 5(29,4%) | 8(44,5%) | 0.358 |
| Yes | 12(70,6%) | 10(55,6%) |  |
| **Fear of gaining weight*** |  |  |  |
| No | 10 (58,8%) | 11 (57,9%) | 0.955 |
| Yes | 7 (41,2%) | 8 (42,1%) |  |
| **Age at onset*** |  |  |  |
| Less than 16 years old | 7 (41,2%) | 12 (63,2%) | 0.187 |
| 17 years old or more | 10 (58,8%) | 7 (36,8%) |  |
| **Number of cigarettes per day**** |  |  |  |
| 10 to 20 cigarettes | 10 (58,8%) | 9 (47,4%) | 0.008 |
| 21 to 30 cigarettes | 1 (5,9%) | 9 (47,4%) |  |
| 31 to 40 cigarettes | 4 (23,5%) | 1 (5,3%) |  |
| More than 40 cigarettes | 2 (11,8%) | 0 (0,0%) |  |
| **Years of active smoking**** |  |  |  |
| 1 to 10 years | 1 (5,9%) | 1 (5,3%) | 0.817 |
| 11 to 20 years | 4 (23,5%) | 5 (26,3%) |  |
| 21 to 30 years | 5 (29,4%) | 5 (26,3%) |  |
| 31 to 40 years | 5 (29,4%) | 3 (15,8%) |  |
| More than 40 years | 2 (11,8%) | 5 (26,3%) |  |
| **Attempts to quit**** |  |  |  |
| never | 4 (23,5%) | 4 (21,1%) | 0.620 |
| Tried but could not | 4 (23,5%) | 2 (10,5%) |  |
| 1 to three times | 7 (41,2%) | 8 (42,1%) |  |
| More than three times | 2 (11,8%) | 5 (26,3%) |  |
| **Thinking of stopping**** |  |  |  |
| No plan to stop | 2 (18,8%) | 3 (15,8%) | 0.885 |
| Yes, in the next six months | 0 (0,0%) | 1 (5,3%) |  |
| Yes, in the next 30 days | 4 (23,5%) | 3 (15,8%) |  |
| Yes, in the next days | 10 (58,8%) | 12 (63,2%) |  |
| Yes, you have already stopped | 1 (5,9%) | 0 (0,0%) |  |
| **How much motivated to stop**** |  |  |  |
| Poorly motivated | 1 (5,9%) | 4 (21,1%) | 0.402 |
| Motivated | 4 (23,5%) | 5 (26,3%) |  |
| Very motivated | 12 (70,6%) | 10 (52,6%) |  |
| **Have you stopped?*** |  |  |  |
| No | 11 (64,7%) | 13 (68,4%) | 0.813 |
| Yes | 6 (35,3%) | 6 (31,6%) |  |
| **Fargestron Scale***** |  |  |  |
| Median (min-max) | 6,5 (1 - 9) | 5,0 (1 - 8) | 0.461 |
| (1) One participant had inconclusive SCID |  |  |  |
| * Chi-Square Test |  |  |  |
| ** Fisher Exact Test |  |  |  |
| *** Mann-Whitney Test |  |  |  |

Table s2 – Participants smoking characteristics in the active and sham groups.
