# Supplementary figures and images for: Effects of Prefrontal Transcranial Direct Current Stimulation and Motivation to Quit in Tobacco Smokers: A Randomized, Sham Controlled, Double-Blind Trial
Source: Front Pharmacol. 2018 Jan 26;9:14. doi: 10.3389/fphar.2018.00014 (PMC5791546; doi:10.3389/fphar.2018.00014)

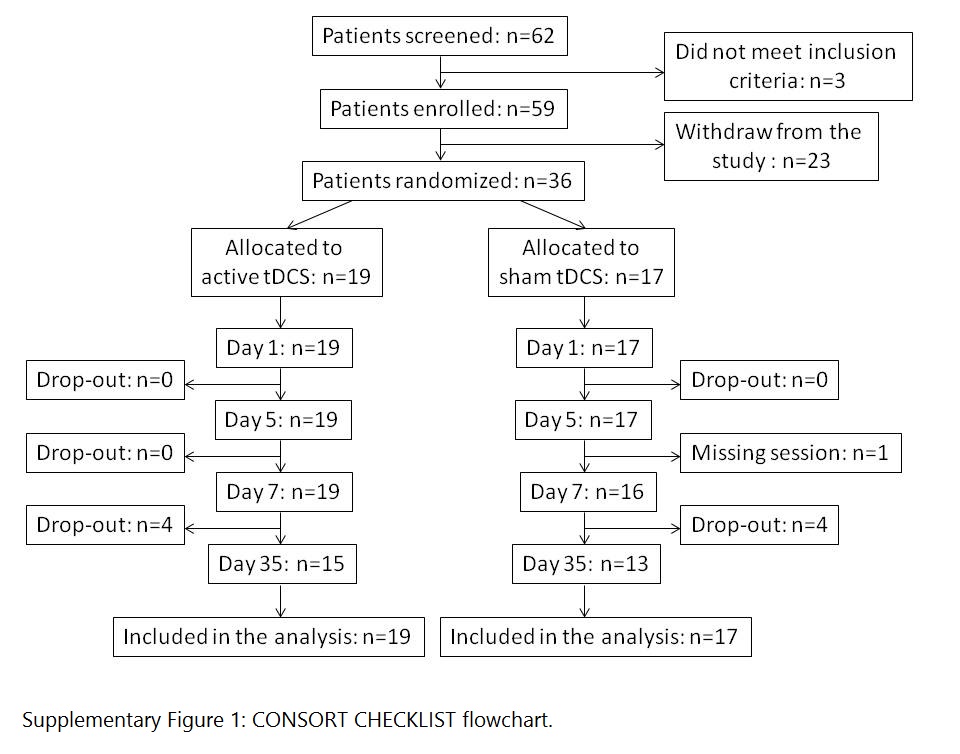

Supplement: Supplementary file 2 [file Image1.jpeg]
